# Supplementary figures and images for: Characterization of transcriptomic changes in the neurovascular unit of Alzheimiers transgenic mouse models using digital spatial profiling
Source: bioRxiv. 2025 May 28:2025.03.03.640886. Preprint. [Version 2] doi: 10.1101/2025.03.03.640886 (PMC12148062; doi:10.1101/2025.03.03.640886)

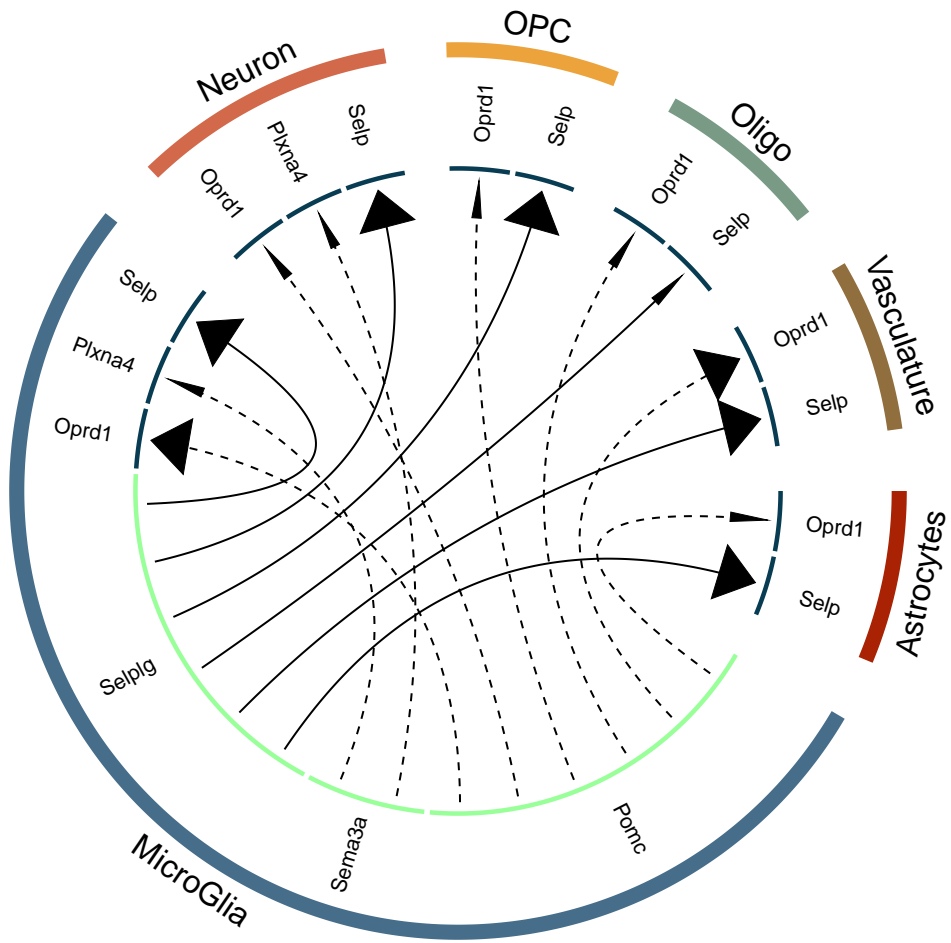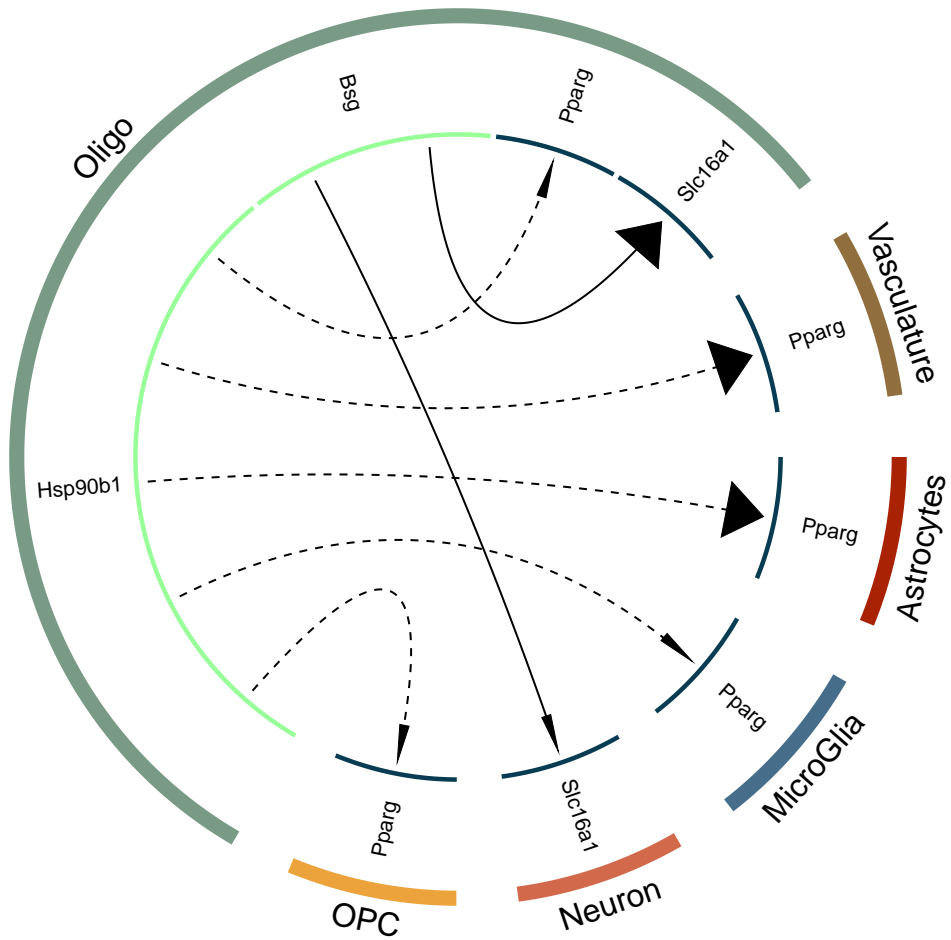

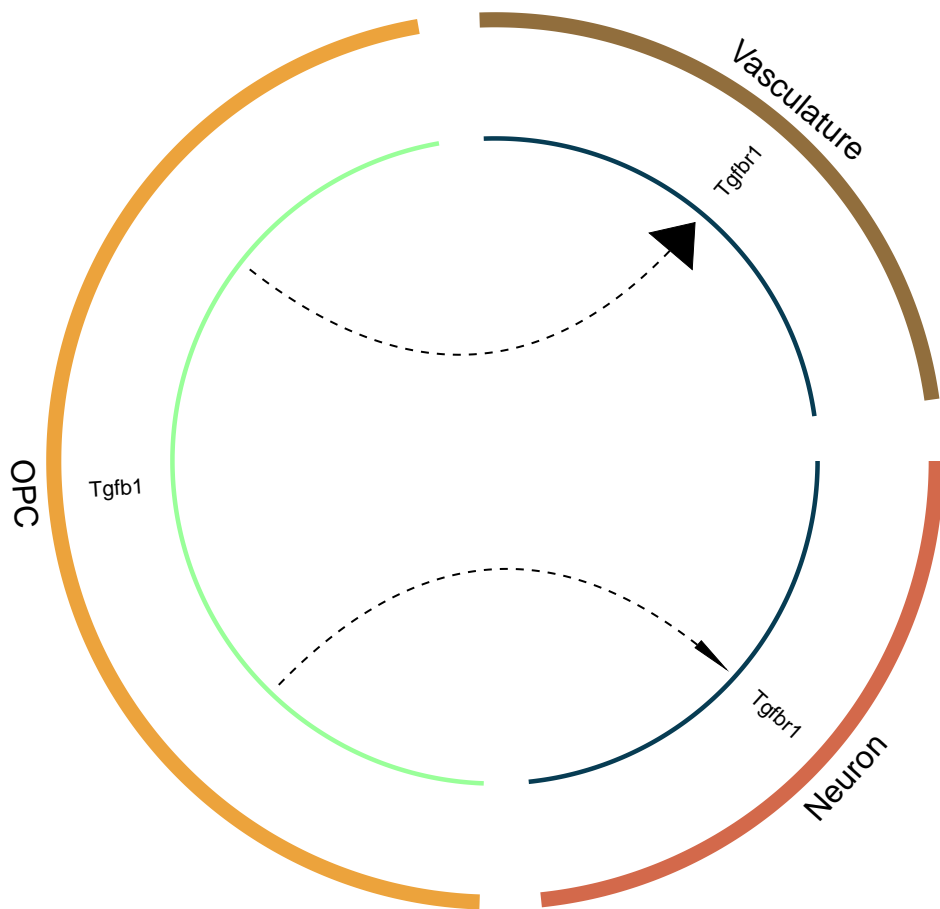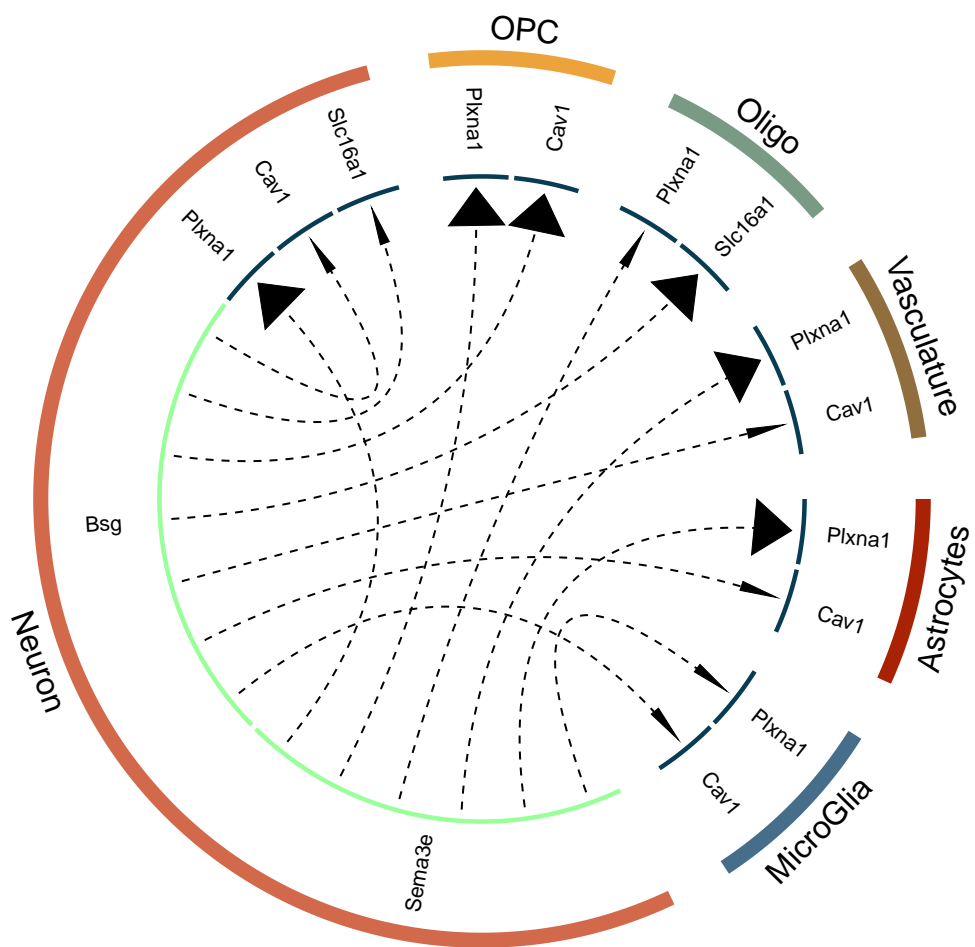

Supplement: Supplement 3 [file media-3.pdf]
